# Supplementary figures and images for: Morphological, histological and transcriptomic mechanisms underlying different fruit shapes in Capsicum spp
Source: PeerJ. 2024 Sep 30;12:e17909. doi: 10.7717/peerj.17909 (PMC11448748; doi:10.7717/peerj.17909)

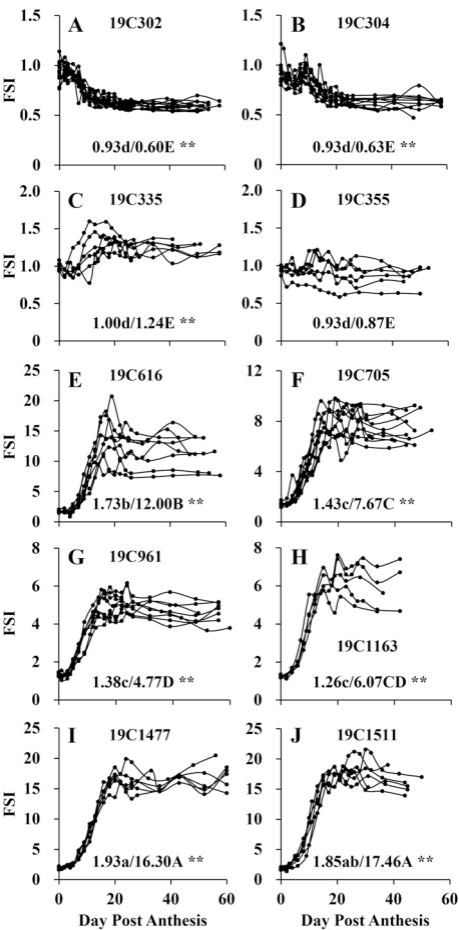

Supplement: Supplemental Information 1 — In each panel, the number in front of the slash indicates the average value of the anthesis ovary shape index, meanwhile, the number behind the slash represents the average value of fruit shape index at the last development stage. Lowercases and capital letters indicate the significance of the HSD test of the anthesis ovary shape index and fruit shape index at the last development stage, respectively. “**” indicates the significant difference between the anthesis ovary shape index and fruit shape index at the last development stage at 0.01 level. [file peerj-12-17909-s001.pdf]
